# Stress Evaluation of Mouse Husbandry Environments for Improving Laboratory Animal Welfare

**DOI:** 10.3390/ani13020249

**Published:** 2023-01-10

**Authors:** Gwang-Hoon Lee, KilSoo Kim, Woori Jo

**Affiliations:** 1 Preclinical Research Center, Daegu-Gyeongbuk Medical Innovation Foundation, Daegu 41061, Republic of Korea; 2 Department of Veterinary Toxicology, College of Veterinary Medicine, Kyungpook National University, Daegu 41566, Republic of Korea

**Keywords:** animal welfare, environmental enrichment, housing, laboratory animals, stress evaluation

## Abstract

**Simple Summary:**

It is well recognized that companionship is important to animals and that they need to be provided with an environment accompanied by materials for enrichment, such as toys. However, few studies have evaluated whether specific environments actually benefit animals. Therefore, we designed various environments for laboratory animals and scientifically evaluated which environments reduced these animals’ stress. We found that an environment with freer air circulation and the provision of enrichment materials reduced animal stress, and no risk or benefit could be determined for the presence or absence of a companion. We do not consider that our results necessarily indicate the lack of a need for a companion, but, rather, the importance of having a good companion. Our results can serve as a meaningful guideline for the creation of suitable environments for laboratory animals.

**Abstract:**

Animal welfare is recognized as essential for the coexistence of humans and animals. Considering the increased demand and interest in animal welfare, many methods for improving animal welfare are being devised, but which method reduces animal stress has not been scientifically verified. Therefore, reducing animal stress by providing a proper breeding environment and environmental enrichment can be the basis for animal study. In this study, stress levels were assessed based on the mouse-breeding environment. We considered that the higher the body weight and the lower the corticosterone concentration, the lower the stress. According to the results, animals in the individual ventilation cages were determined to have lower serum cortisol concentrations, while the body weight of the animals was increased when in individual ventilation cages compared with individual isolated cages and when providing environmental enrichment compared with group breeding or not providing environmental enrichment. The results provide appropriate guidelines for improving laboratory animal welfare.

## 1. Introduction

With the recent increasing interest in animal ethics, there has been a growing focus within the international laboratory animal research community on improving the housing environment and welfare of laboratory animals [1]. Although related laws and systems have been continuously strengthened in the field of laboratory animal studies, issues regarding ethical relations between humans and animals in bioscience research laboratories remain. Since the United States of America first enacted the laboratory animal welfare act in 1966, laws related to animal welfare have continued to be strengthened [2]. The Animal Welfare Act: From Enactment to Enforcement of the Europe Union (E.U) also extensively revised laws regarding animal welfare, including for laboratory animals, since 1974 [3]. The Ministry of Agriculture, Food, and Rural Affairs (MAFRA, Republic of Korea) announced a five-year comprehensive plan for animal welfare to raise public awareness of and sympathy for animal protection and welfare, and to enhance animal laboratory animal ethics by reinforcing the function of the Institutional Animal Care and Use Committee (IACUC). Furthermore, recent debates on laboratory animal welfare have been conducted at the National Assembly.

In addition, unlike domestic companion animals, laboratory animals are limited to a fixed space for the entire experimental period, so laboratory animals are more exposed to stress from the housing environment than companion animals. This constrained environmental condition can cause stress in animals, which affects their physiological indicators and can change the results of experiments. Studies associated with laboratory animal behavioral analysis have also reported that housing conditions, such as a socially isolated environment, group size, or cage size, are an important factor influencing animal behavior [4,5,6,7]. Therefore, to obtain reliable experimental results in laboratory animal research, considering animal welfare is important, as it can alleviate the stress experienced by laboratory animals, affecting the experimental results [8]. Furthermore, policies and practices with respect to laboratory animal housing, husbandry, and quality care can enhance animal welfare [9,10,11]. A previous study demonstrated that the welfare of laboratory animals contributes to improving the quality of life for the people involved in animal research facilities. Animal welfare staff showed a more positive correlation with a professional quality of life than researchers who were reported to perceive animal stress/pain [10]. 

Laboratory animals are used in a variety of research fields, and stress induced during experiments can change the background data. Stress is defined as the body’s nonspecific response to external stimuli, environmental demands, or stimuli beyond the body’s ability to cope [12]. Vladimir K et al. reported that stress causes changes in the limbic–hypothalamic–pituitary–adrenal (LHPA) neuroendocrine axis, De Kloet ER et al. described that stress induced structural and functional change in the limbic brain, and B Olivier demonstrated that stress increases the body temperature of laboratory animals [13,14,15]. 

Most studies on stress involve experiments with artificially applied stress, including, among others, repeated social defeat stress (RSDS), electric shock, wire netting, and repeated stress [16,17,18,19,20]. In addition, previous studies on the efficacy of newly developed drugs have been based on either the use of an artificially induced stress model [16,20] or measuring the hormonal changes caused by different levels of stress exposure [21]. The interest and demand regarding the welfare of zoo animals and laboratory animals is also elevated because of their stereotypic behavior affected by limited breeding space. Many methods for improving their behavior are devised with environmental enrichment to respect their natural habits [22]. However, studies on stress levels after exposure to various housing environments with regard to the improvement of laboratory animal welfare for animals that live in limited space are lacking. Furthermore, it is necessary to evaluate environmental effects on the stress of laboratory animals depending on the type of cage and/or social isolation.

Stress–response hormones include cortisol and corticosterone. Cortisol is widely considered in studies on large laboratory animals such as beagles [23,24], whereas for smaller animals, such as rodents, corticosterone is the more important glucocorticoid in responding to stress exposure [19].

Therefore, in this study, we aimed to evaluate the changes in the serum corticosterone concentration and body weight as stress indicators according to the presence or absence of environmental enrichment (E.E), different types of cages (individually ventilated cages (IVC) or individually isolated cages (ISO)), and social isolation stress (single breeding or group breeding). It is hoped that these results will be useful for enhancing the environmental conditions for laboratory animals and improving animal welfare by reducing stress on laboratory animals throughout the entire experimental period. 

## 2. Materials and Methods

### 2.1. Animals and Husbandry

Four-week-old CrljOri:CD1 (ICR) male mice were purchased from Orient Bio (Seongnam, KyungKi, Korea). The animal experiments were reviewed and approved by the Institutional Animal Care and Use Committee at the Daegu Gyeongbuk Medical Innovation Foundation (K-MEDI hub) (approved IACUC Number: DGMIF-20032407, approved date: 23 March 2020), and the animals were maintained in a facility accredited by the Ministry of Food and Drug Safety in Korea as a Korean Excellent Laboratory Animal Facility (KELAF), and by the Association for Assessment and Accreditation of Laboratory Animal Care International (#001796). The animals were fed an autoclaved pellet diet (SAFE+40RMM; SAFE Diets, Augy, France) and provided with drinking water ad libitum. The animals were housed in environmental conditions with a temperature of 22 ± 1 °C, 50 ± 10% humidity, illumination at 150–300 Lux, and a breeding room ventilation cycle of 10–20 times/h. All of the animals were monitored every day and there were no mortality injuries or clinical signs. Cages, shavings, and fresh enrichment materials were exchanged once a week.

### 2.2. Experimental Design

**Figure 1:**
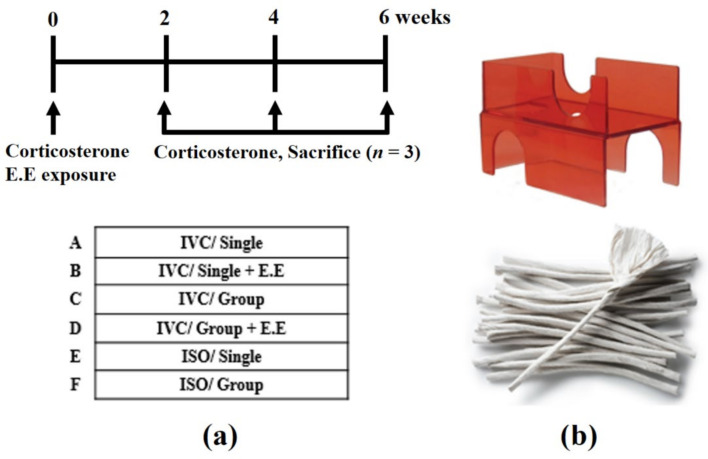
(**a**) Experimental design; (**b**) representative photographs of environmental enrichment (harbor mouse retreat, top; diamond twist, bottom).

The experimental design is shown in Figure 1a. The mice were divided into six groups (nine mice/group) after an initial week of acclimatization, as follows: (A) IVC/Single; (B) IVC/Single + (Environmental Enrichment) E.E; (C) IVC/Group (Group: three mice in one cage); (D) IVC/Group + E.E; (E) ISO/Single; and (F) ISO/Group. Both cage systems (Tecniplast, Buguggiate, Varese, Italy), IVC (Cat No: GM500) and ISO (Cat No. ISO cage-N), had an air-circulation system in each cage, with the main difference being whether room air outside the cage was allowed to enter into the cage with mice inside. In the IVC cage, the air in the cage is not only circulated by the automatic blower system, but room air outside the cage also enter into the cage. Room air outside the cage can enter through the HEPA filter (size: 141 mm × 170 mm, efficiency: 0.3 micrometer of the particle at 99.5%) on the IVC lid, while in the ISO cage (size: 73 mm × 73 mm × 24 mm, efficiency: 0.3 micrometer of the particle at 99.97%), room air outside the cage is completely blocked and air in the cage is forcibly circulated only by the automatic blower system. Therefore, the air in the IVC cage is circulates better than the air in the ISO cage.

Two environmental enrichment (E.E) materials were used simultaneously, the first being a harbor mouse retreat (Figure 1b top, Cat No. K3583, Bio-serv, Frenchtown, NJ, USA) and the second being a diamond twist (Figure 1b bottom, Envigo, Madison, WI, USA), which were provided to groups B and D until the end of the experiment. The harbor mouse retreat and the diamond twist were selected as the E.E materials as the standard environmental enrichment required and an additional enrichment, respectively, with reference to the IACUC policy of the University of California, Irvine (Figure 1).

### 2.3. Preparation of the Blood Serum and Corticosterone Assay

In the present study, blood collection was performed once every 2 weeks, with three mice in each group. The mice were anesthetized using isoflurane at 17:00–18:00 p.m., and blood was rapidly collected to minimize the stress caused by the anesthesia process, and euthanasia was performed by exsanguination under anesthesia. Because of the slight possibility of survival, cervical dislocation was performed under anesthesia. As it is painful to live after bloodletting, cervical dislocation was performed to block the weak possibility of living under anesthesia. Blood was collected from the abdominal vein (approximately 600 μL) in serum-separating tubes (SST tube, Becton, Dickinson and Company, Franklin Lakes, NJ, USA) and was centrifuged (3000 rpm, 10 min, 4 °C) to separate the serum. 

Triplicate serum corticosterone assays were conducted using an ELISA kit (Cat No. K014, Arbor assays, Ann Arbor, MI, USA), and the optical density (OD) was read using a synergy H4 microplate reader (BioTek Instruments, Inc., Winooski, VT, USA).

### 2.4. Statistical Analysis

Statistical significance was determined using GraphPad Prism 8 (GraphPad Software Inc., San Diego, CA, USA). All the data are presented as mean ± standard deviation (SD) and passed tests for normality. Two-way ANOVA with Bonferroni’s multiple comparisons test and unpaired t-test were used.

## 3. Results

### 3.1. Body Weight

**Figure 2:**
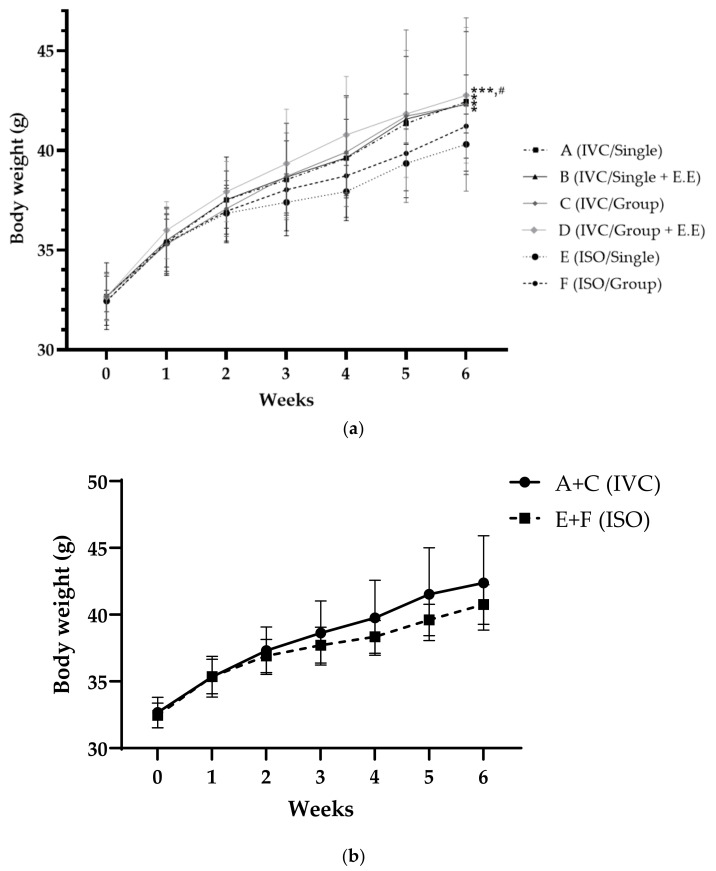
Body weight changes in (**a**) each group and (**b**) groups A + C and E + F (* *p* < 0.05 and *** *p* < 0.001 compared to E, # *p* < 0.05 compared to F), (0~2 weeks: *n* = 9/group, 2~4 weeks: *n* = 6/group, 4~6 weeks: *n* = 3/group).

After an initial week of acclimatization, the body weight was measured weekly until euthanasia. During the experiment period, group D had the highest average body weight, followed by groups A, C, B, F, and E. There was a significant difference (*p* < 0.05) between groups D and F, and E was significantly different from A, B, C, and D (Figure 2).

### 3.2. Concentrations of Serum Corticosterone

**Figure 3:**
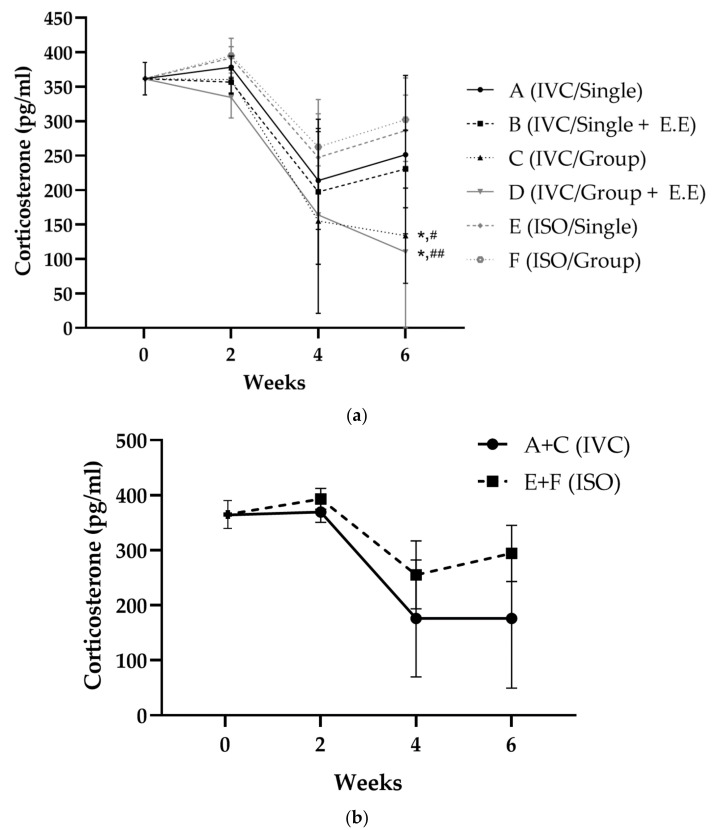
(**a**) Corticosterone concentration in the serum change for each group; (**b**) corticosterone concentration in the serum change for groups A + C and E + F (* *p* < 0.05 compared with E, # *p* < 0.05 compared with F, ## *p* < 0.01 compared with F).

From 2 to 4 weeks, the serum corticosterone concentrations decreased in all groups; from 4 to 6 weeks, the concentrations decreased in groups C and D, which had been group-reared, and increased slightly again in the remaining groups. The serum corticosterone concentration showed the order of F, E, A, B, C, and D at week 6, with a significant difference (*p* < 0.05) between groups C and E, C and F, D and E, and D and F (Figure 3).

### 3.3. Concentrations of Serum Corticosterone/Body Weight

**Figure 4:**
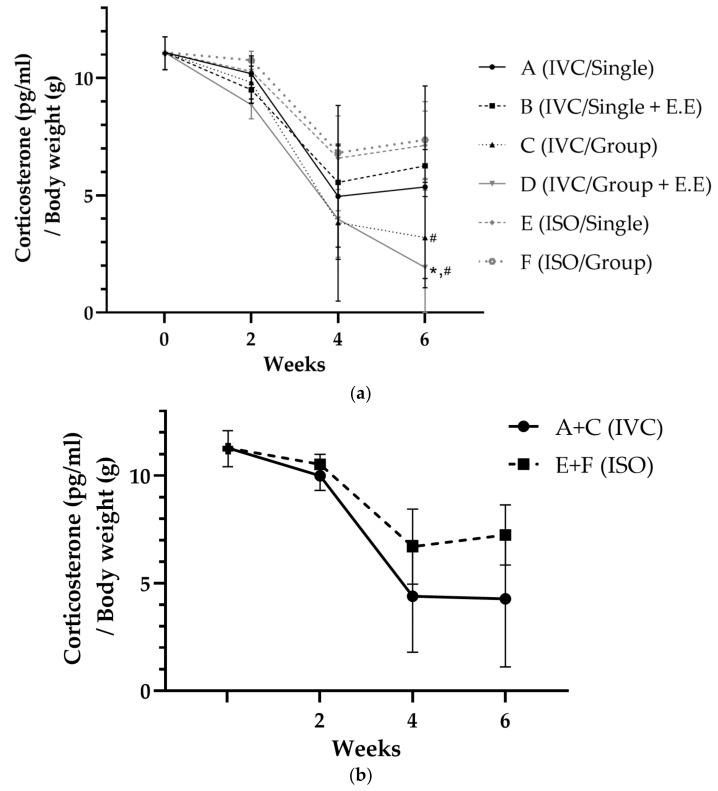
Corticosterone concentration in the serum/body weight change for (**a**) each group and (**b**) groups A + C and E + F (* *p* < 0.05 compared with E, # *p* < 0.05 compared with F).

The relative corticosterone concentration (corticosterone/body weight) had an overall similar tendency to the serum corticosterone level. The only observed difference was that the order of A and B was reversed at 2 and 4 weeks. In addition, there was a significant (*p* < 0.05) difference between groups C and F, D and E, and D and F (Figure 4).

### 3.4. Overall Analysis

The IVC cage/Group + E.E group mice had the highest body weight and the lowest corticosterone concentration on average, the ISO cage/Single group mice had the lowest body weight, and the ISO cage/Group mice had the lowest corticosterone concentration on average. All IVC cage mice weighed more and had lower corticosterone concentrations than all ISO cage mice on average. The mice from groups with E.E had a higher body weight and lower corticosterone concentration than those of groups without E.E on average (IVC cage/Group + E.E vs. IVC cage/Group, IVC cage/Single + E.E vs. IVC cage/Single) (Table 1).

## 4. Discussion

In this study, we created six different environmental conditions for mice at the Preclinical Research Center (PRC), K-MEDI hub to evaluate the levels of stress. 

Studies have been carried out on the effectiveness of E.E [24,25,26,27] at preventing oxidative injury and restoring cholinergic neurotransmission in cognitively impaired aged rats [25], as well as in alleviating the behavioral changes in a mouse model of post-traumatic stress disorder [28]. In addition, studies regarding the relationship between housing conditions, such as single housing or grouped housing, and behavioral phenotypes have been conducted [29]. Both isolation and environmental enrichment have fundamental effects on mouse behavior and should be considered in the course of experimental design with stress-related animal models and in animal welfare assessment. 

In this study, body weight can be an indicator of stress level, as it is generally known that stress can cause the amount of feed intake and body weight to decrease [16,17,18]. This study reports that animals exposed to stress might have a decrease in the body weight gain rate. Based on the results of body weight for the groups, group D (IVC cage/Group + E.E), in which body weight increased the most, was less stressed, and group E (ISO cage/Single), with the least body weight gain, represented the more stressful environment (Table 1). Corticosterone in the blood is a factor widely known to increase when a mouse is exposed to stress [19]. Therefore, based on the results of the serum corticosterone concentration, the mice of group D (IVC cage/Group + E.E), with the lowest concentration, were the least stressed, and those of group F (ISO cage/Group), with the highest concentration, were the most stressed. Overall, the concentration of serum corticosterone was the highest in the second week and then decreased. The results of the relative serum corticosterone (corticosterone/body weight) were generally similar to the serum corticosterone results, with the only change being in the order of group A (IVC/Single) and group B (IVC/Single + E.E). This slight change means that stress had no biased effect on either body weight or corticosterone blood levels. The serum corticosterone concentrations were similar between weeks 0 and 2, but the relative corticosterone levels decreased at week 2. We judged that this was due to the maintained corticosterone concentration contrary to the increased body weight.

Based on these results, group D (IVC/Group + E.E) was the group with the highest body weight and lowest corticosterone concentration, on average, and it was thus the group that was the least stressed. 

Furthermore, the groups with E.E showed a less stressful environment than the groups without E.E. However, there was no difference in body weight or corticosterone concentration between group breeding and single breeding. Moreover, it was difficult to judge the results of the relationship between the group housing and single housing conditions, and we did not consider a sufficient number of individuals in our experiment. In addition, opinion is divided regarding the factors attributed to social ranking, which can also be a hindrance when judging the results. Benaroya-Milshtein and Hollander et al. reported that E.E reduces anxiety and weakens stress reactions in mice, while Chapillon et al. reported that environmental energy in mice reduces anxiety [27,30]. Although the mouse is a social animal, it is judged that there can be differences among animals exposed to the same stress due to differences in hierarchy that are determined by the group. Both group and single breeding are known to have pros and cons, and the various experiments show conflicting results [9]. Liu and Wang et al. reported that single mice had a reduced concentration of corticosterone, while Kamakura and Kovalainen et al. reported that group-housed mice had higher levels of corticosterone than single-house mice, indicating that those mice living in groups had higher levels of stress [31,32]. On the other hand, Norman et al. reported that single mice had a significantly reduced memory, and according to a study by Kamal et al., the level of LTP injury was increased in the hippocampus of C57BL/6J mice in the single-breeding group, which also had higher blood corticosterone concentrations [33,34].

In addition, in this study, the average weight of the ISO cage mice was lower than that of the IVC cage mice, while the blood corticosterone concentration was higher on average, so it was judged that the stress was higher because the ISO cage has limited air circulation. The graph pattern for the change in corticosterone value and corticosterone/body weight value was not significantly different, even if the ranking of the average value for each factor was slightly different. 

Furthermore, stress effects may differ depending on the mouse strain, so further studies are needed on stress using diverse strains and species of animals with disease as models [15]. In addition, with the growing interest in animal welfare, stress assessments could be expanded to abandoned animals or industrial animals [26]. 

Full accreditation for The Association for Assessment and Accreditation of the Laboratory Animal Care International (AAALAC-i) is a recognized certification awarded by a nonclinical study institution that encourages the humane treatment of animals in the field of science by maintaining a high level for laboratory animal care and use [35]. In addition to this full AAALAC-i accreditation, the PRC at the K-MEDI hub is devising and continuing to develop ways to promote the welfare of laboratory animals. 

These results suggest that IVC cages are preferable to ISO cages and that providing E.E would be more effective for relieving stress. However, the appropriate composition of different animals raised in the same cage is needed because it may influence whether stress is more active. The results of this study are helpful by serving as appropriate guidelines for the management and operation of laboratory animal breeding and the welfare of laboratory animals. 

Animals are originally organisms that live in nature, but laboratory animals inevitably live in a limited space. It is difficult to derive reproducible and reliable experimental results from psychologically fatigued animals [36]. We judged that the environmental conditions that reduced stress in this experiment were conditions close to the animal’s natural habit. Therefore, our study suggests that environmental enrichment that respects their natural habits should be provided for reliable animal test results. Ultimately, our society already knows that animals are not voluntarily subjected to experiments. We believe that any facility that raises animals should be induced to change to a facility that values the autonomy of animals, and this study can serve as a basis for inducing that change.

Future studies regarding the effects of stress on animals should include behavioral assessments. Luo et al. demonstrated that displacement movement can be predicted by evaluating random behavior permutation in ungulates, and they reported that the differences could be found when comparing the isolated environment considered as stressful and an enriched environment considering their natural habits [37]. Considering their report, we judge that meaningful behavioral data can be achieved if we conduct a study by applying our experimental environmental conditions on mice as a laboratory animal.

## 5. Conclusions

We have scientifically confirmed a reduction in stress with environmental enrichment, or for animals raised together with other animals, or in a well-ventilated environment in mice. 

With the results of this study, we determine that animals raised in limited space such as an animal research facility should be provided with environmental enrichment, a well-ventilated environment. Because the environments that reduce stress are thought as a result of the natural habits of animals, we should devise various methods to respect their natural habits as much as possible, and evaluate whether these methods reduce their stress. 

This data will be guideline to all researchers who work in a laboratory animal facility. Laboratory animals with improved environmental enrichment can achieve reliable scientific results.

## Figures and Tables

**Table 1:** Overall analysis of body weight, serum corticosterone concentration, and corticosterone concentration/body weight.

Stress Level	Less Stressful	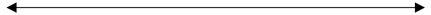				More Stressful
Body weight (g)	1st	2nd	3rd	4th	5th	6th
	IVC cage	IVC cage	IVC cage	IVC cage	ISO cage	ISO cage
	Group	Single	Single	Group	Group	Single
	E.E *	E.E *				
Corticosterone concentration in serum (pg/mL)	6th	5th	4th	3rd	2nd	1st
	IVC cage	IVC cage	IVC cage	IVC cage	ISO cage	ISO cage
	Group	Group	Single	Single	Single	Group
	E.E *		E.E *			
Corticosterone concentration/body weight	6th	5th	4th	3rd	2nd	1st
	IVC cage	IVC cage	ISO cage	IVC cage	ISO cage	ISO cage
	Group	Group	Single	Single	Single	Group
	E.E *			E.E *		

* E.E: environmental enrichment.

## Data Availability

The data presented in this study are available on request from the corresponding author.
